# Extracorporeal Shock Wave-Supported Adipose-Derived Fresh Stromal Vascular Fraction Preserved Left Ventricular (LV) Function and Inhibited LV Remodeling in Acute Myocardial Infarction in Rat

**DOI:** 10.1155/2018/7518920

**Published:** 2018-10-17

**Authors:** Pei-Hsun Sung, Tsung-Cheng Yin, Christopher Glenn Wallace, Kuan-Hung Chen, Pei-Lin Shao, Fan-Yen Lee, Cheuk-Kwan Sun, Jiunn-Jye Sheu, Yung-Lung Chen, Mostafa Mohammad Omran, Sheng-Ying Chung, Ching-Jen Wang, Mel S. Lee, Hon-Kan Yip

**Affiliations:** ^1^Division of Cardiology, Department of Internal Medicine, Kaohsiung Chang Gung Memorial Hospital and Chang Gung University College of Medicine, Kaohsiung 83301, Taiwan; ^2^Center for Shockwave Medicine and Tissue Engineering, Kaohsiung Chang Gung Memorial Hospital and Chang Gung University College of Medicine, Kaohsiung, Taiwan; ^3^Department of Orthopedics, Kaohsiung Chang Gung Memorial Hospital and Chang Gung University College of Medicine, 83301 Kaohsiung, Taiwan; ^4^Department of Plastic Surgery, University Hospital of South Manchester, Manchester, UK; ^5^Department of Anesthesiology, Kaohsiung Chang Gung Memorial Hospital and Chang Gung University College of Medicine, Kaohsiung 83301, Taiwan; ^6^Department of Nursing, Asia University, Taichung 41354, Taiwan; ^7^Division of Thoracic and Cardiovascular Surgery, Department of Surgery, Kaohsiung Chang Gung Memorial Hospital and Chang Gung University College of Medicine, Kaohsiung 83301, Taiwan; ^8^Division of Cardiovascular Surgery, Department of Surgery, Tri-Service General Hospital, National Defense Medical Center, Taiwan; ^9^Department of Emergency Medicine, E-Da Hospital, I-Shou University School of Medicine for International Students, Kaohsiung 82445, Taiwan; ^10^Department of Cardiology, National Heart Institute, Egypt; ^11^Institute for Translational Research in Biomedicine, Kaohsiung Chang Gung Memorial Hospital, Kaohsiung, Taiwan; ^12^Department of Medical Research, China Medical University Hospital, China Medical University, Taichung 40402, Taiwan

## Abstract

This study tested the hypothesis that extracorporeal shock wave- (ECSW-) assisted adipose-derived stromal vascular fraction (SVF) therapy could preserve left ventricular ejection fraction (LVEF) and inhibit LV remodeling in a rat after acute myocardial infarction (AMI). Adult male SD rats were categorized into group 1 (sham control), group 2 (AMI induced by left coronary artery ligation), group 3 [AMI + ECSW (280 impulses at 0.1 mJ/mm^2^, applied to the chest wall at 3 h, days 3 and 7 after AMI), group 4 [AMI + SVF (1.2 × 10^6^) implanted into the infarct area at 3 h after AMI], and group 5 (AMI + ECSW-SVF). In vitro, SVF protected H9C2 cells against menadione-induced mitochondrial damage and increased fluorescent intensity of mitochondria in nuclei (*p* < 0.01). By day 42 after AMI, LVEF was highest in group 1, lowest in group 2, significantly higher in group 5 than in groups 3 and 4, and similar between the latter two groups (all *p* < 0.0001). LV remodeling and infarcted, fibrotic, and collagen deposition areas as well as apoptotic nuclei exhibited an opposite pattern to LVEF among the groups (all *p* < 0.0001). Protein expressions of CD31/vWF/eNOS/PGC-1*α*/*α*-MHC/mitochondrial cytochrome C exhibited an identical pattern, whilst protein expressions of MMP-9/TNF-*α*/IL-1*β*/NF-*κ*B/caspase-3/PARP/Samd3/TGF-*β*/NOX-1/NOX-2/oxidized protein/*β*-MHC/BNP exhibited an opposite pattern to LVEF among five groups (all *p* < 0.0001). Cellular expressions of CXCR4/SDF-1*α*/Sca-1/c-Kit significantly and progressively increased from groups 1 to 5 (all *p* < 0.0001). Cellular expression of *γ*-H2AX/CD68 displayed an opposite pattern to LVEF among the five groups (all *p* < 0.0001). In conclusion, ECSW-SVF therapy effectively preserved LVEF and inhibited LV remodeling in rat AMI.

## 1. Introduction

Even undergoing rapid reperfusion therapy [1–5], acute myocardial infarction (AMI) causes muscle death then pump failure. Accordingly, tissue regeneration may have a therapeutic role for AMI patients. Abundant data supports that stem cell therapy can improve ischemia-related organ dysfunction, particularly for cardiovascular diseases, including AMI [6–10]. Most experimental or clinical trials indicate that an adequate number of stem cells are essential for ischemia-related organ dysfunction to be treated successfully [6, 11–13]. Stem cell culture and expansion are therefore necessary [14–16], but these take too long for AMI patients for whom rapid treatment is critical. Accordingly, an innovative method that could provide adequate stem cells quickly without cell culture and be effective as a treatment for AMI would be of great clinical importance.

It was recently reported that treatment with adipose-derived fresh stromal vascular fraction (SVF) containing primitive stem cells, which could be utilized immediately and without need for cell culture, accelerated wound healing through angiogenesis and anti-inflammation [17, 18]. Indeed, adipose-derived fresh SVF-enriched heterogeneous populations of undifferentiated, mononucleated elements, as based on cell surface antigens within those multipotent tissues [18], are emerging as an easy and safe way to treat various diseases [18–20]. However, the therapeutic potential of SVF on cardiac function following AMI has seldom been investigated [21].

Extracorporeal shock wave (ECSW) has successfully treated chronic ischemic cardiovascular disease and acute ischemic stroke in experimental models [21–28]. ECSW therapy appears to improve ischemia-related organ dysfunction mainly through enhancing angiogenesis, upregulating SDF-1*α* expression, recruiting endothelial progenitor cells, and suppressing inflammation, generation of oxidative stress, and cell apoptosis [21, 22, 24, 28]. We have further established that combined therapy with ECSW and bone marrow-derived mesenchymal stem cells was superior to either alone for improving LVEF, reducing infarct size, and inhibiting LV remodeling [24].

Based on these studies [17–28], we tested the hypothesis that combined therapy using ECSW and SVF could be superior to either alone for improving LVEF and inhibiting LV remodeling in a rodent model of AMI.

## 2. Materials and Methods

### 2.1. Ethics

All animal procedures were approved by the Institute of Animal Care and Use Committee at Kaohsiung Chang Gung Memorial Hospital (Affidavit of Approval of Animal Use Protocol No. 2016012703) and performed in accordance with the Guide for the Care and Use of Laboratory Animals.

Animals were housed in an Association for Assessment and Accreditation of Laboratory Animal Care International- (Frederick, MD, USA) approved animal facility in our hospital with controlled temperature and light cycles (24°C and 12/12 light cycle).

### 2.2. Animal Grouping

Adult male Sprague Dawley rats (*n* = 48) weighing 325–350 g (Charles River Technology, BioLASCO, Taiwan) were categorized into five groups: sham-operated control (SC; the chest wall opened and the left anterior descending artery (LAD) was identified), AMI (induced by ligating the left coronary artery), AMI + ECSW (i.e., AMI-ECSW) (280 impulses at 0.1 mJ/mm^2^ applied to the chest wall at 3 h, 3 days and 7 days after AMI induction), AMI + SVF [(i.e., AMI-SVF) (1.2 × 10^6^) by implantation into the infarcted area at 3 h after AMI induction], and AMI + ECSW + SVF (AMI-ECSW-SVF).

The stem cell doses utilized in the present study were based on our previous reports [9, 29, 30], as was the energy of ECSW application to groups AMI-ECSW and AMI-ECSW-SVF [28].

Six animals per group were used to identify infarcted, fibrotic, and collagen deposition areas in cardiac cross-sections. Six animals per group were utilized for molecular-cellular studies.

### 2.3. Animal Model of AMI

The procedure and protocol for AMI induction were as previously reported [9, 30]. All animals were anesthetized (inhalational 2.0% isoflurane) supine on a warming pad at 37°C, and the heart was exposed via a left thoracotomy under sterile conditions. Sham-operated rats (SC) received thoracotomy alone; AMI in other groups was induced by left coronary artery ligation 3 mm distal to the margin of the left atrium with 7-0 prolene. The thoracotomy was then closed after SVF implantation for different sites in the ischemic area, and the animals recovered in a portable animal intensive care unit (ThermoCare®) for 24 hours.

### 2.4. Functional Assessment by Echocardiography

Transthoracic echocardiograms (Vevo 2100, VisualSonics, Toronto, Ontario, Canada) were standardized as previously reported [22, 24, 30] and performed in animals from each group prior to and at day 60 after doxorubicin treatment by a veterinary cardiologist blinded to the experimental design. Left ventricular internal dimensions [i.e., left ventricular end-systolic diameter (LVESd) and left ventricular end-diastolic diameter (LVEDd)] were measured at mitral valve and papillary levels of the left ventricle, as per the American Society of Echocardiography (Morrisville, NC) leading-edge methodology, using at least three consecutive cardiac cycles. Left ventricular ejection fraction (LVEF) was calculated as follows: LVEF (%) = [(LVEDd^3^−LVESd^3^)/LVEDd^3^] × 100%.

### 2.5. Procedure and Protocol for Isolation of Adipose-Derived Fresh SVF

The procedure and protocol for preparing SVF were performed as previously reported [21]. Rats in groups 4 and 5 were anesthetized with inhalational isoflurane 3 h prior to AMI induction. Both inguinal regions were clipped and prepared with 10% povidone iodine; the inguinal fat pads were removed, and a 1 × 1 × 1 cm (1 cm^3^) of adipose tissue was excised from each. We pooled all cells from the same rats to make a master batch of SVF. The fragmented tissues were incubated with 0.1% collagenase (collagenase from Clostridium histolyticum C0130, Sigma 1) and kept in a slow shaking water bath at 37°C for 60 min. The cell suspension was centrifuged twice at 1300 rpm (260g) for 5 min. The supernatant containing mature adipocytes was removed. The precipitate was passed through a 100 *μ*m mesh filter and used as SVF. Viable cells were counted (Thoma slide) by adding trypan blue to SVF. There were approximately 4 × 10^6^/ml viable cells.

### 2.6. Procedure and Protocol of H9C2 Cells Cocultured with Menadione and SVF

Freshly isolated adipose tissue from rat was minced with dissecting scissor and incubated with 1 mg/ml collagenase A type II at 37°C for 30 minutes. After reaction was stop with stop buffer (growth medium with 20% FBS), adipose tissue extract (i.e., contained SVF component) was filtrated with a cell strainer and centrifuged at 500xg for 5 minutes. The red blood cells in the cell pellets were removed by incubation with ACK (ammonium-chloride-potassium) lysing buffer for 1 minute. Following PBS washing, cells were collected by centrifugation at 500xg for 5 minutes and cultured at 37°C under 5% CO_2_ in 1 well of cell culture insert (8 *μ*m pore size) containing 2 ml of low glucose Dulbecco's modified Eagle's medium supplemented with 10% fetal bovine serum, 100 *μ*g/ml streptomycin, and 100 units/ml penicillin. After 24 hr incubation, the cell culture insert was replaced to the well of H9C2. Following 24 hr coculture, the cell culture insert was removed, and menadione (25 *μ*M) treatment was performed.

### 2.7. Transfection of Cells with Plasmid MT-EGFP for Identification of Fusion and Fission after Menadione Treatment

Human cytochrome C oxidase sub-VIII mitochondrial targeting sequence (MTS) 1-29 amino acids were fused with EGFP fluorescence protein (MT-EGFP) by a PCR machine. The PCR product was constructed into a pcDNA3.1 His B vector. Transient transfection of cells with plasmid was performed with Lipofectamine 3000 (Invitrogen, Life Technologies, Carlsbad, CA, USA) according to the manufacturer's instructions with minimal modifications. Cells were replated 24 h before transfection at a density of 1.0 × 10^6^ H9C2 cells in 2 ml of fresh culture medium in a 3.5 cm plastic dish. For transfection procedure, 4 *μ*l of Lipofectamine 3000 was incubated with 2 *μ*g of plasmid and 5 *μ*l P3000 reagent at room temperature. Cells were then incubated at 37°C in a humidified atmosphere of 5% CO_2_ before fixation.

### 2.8. Western Blot Analysis

As previously reported [31, 32], equal amounts (50 *μ*g) of protein extracts were loaded and separated by SDS-PAGE using acrylamide gradients. After electrophoresis, the separated proteins were transferred electrophoretically to a polyvinylidene difluoride (PVDF) membrane (Amersham Biosciences). Nonspecific sites were blocked by incubating the membrane in a blocking buffer [5% nonfat dry milk in T-TBS (TBS containing 0.05% Tween 20)] overnight. The membranes were incubated with the indicated primary antibodies [mitochondrial (mit-) Bax (1 : 1000, Abcam), cleaved caspase 3 (c-Csp3) (1 : 1000, Cell Signaling), cleaved poly(ADP-ribose) polymerase (PARP) (1 : 1000, Cell Signaling), phosphorylated- (p-) Smad3 (1 : 1000, Cell Signaling), transforming growth factor- (TGF-) *β* (1 : 500, Abcam), interleukin- (IL-) 1*β* (1 : 1000, Cell Signaling), matrix metalloproteinase- (MMP-) 9 (1 : 3000, Abcam), tumor necrosis factor- (TNF-) *α* (1 : 1000, Cell Signaling), nuclear factor- (NF-) *κ*B (1 : 1000, Abcam), CXCR4 (1 : 1000, Abcam), stromal cell-derived factor- (SDF-) 1*α* (1 : 1000, Cell Signaling), endothelial nitric oxide synthase (eNOS) (1 : 1000, Abcam), vascular endothelial cell growth factor (VEGF) (1 : 1000, Abcam), CD31 (1 : 1000, Abcam), mitochondrial cytochrome C (mit-Cyto C) (1 : 1000, BD Biosciences), cytosolic cytochrome C (cyt-Cyto C) (1 : 1000, BD Biosciences), brain natriuretic peptide (BNP) (1 : 600, Abcam), myosin heavy chain- (MHC-) *α* (1 : 300, Santa Cruz), MHC-*β* (1 : 1000, Santa Cruz), NOX-1 (1 : 1500, Sigma), NOX-2 (1 : 1000, Sigma), oxyblot (1 : 100, Millipore), peroxisome proliferator activated receptor-*γ* coactivator 1 *α* (PGC-1*α*) (1 : 1000, Abcam), and actin (1 : 10,000, Chemicon)] for 1 hour at room temperature. Horseradish peroxidase-conjugated anti-rabbit immunoglobulin IgG (1 : 2000, Cell Signaling) was used as a secondary antibody for one-hour incubation at room temperature. Immunoreactive bands were visualized by enhanced chemiluminescence (ECL; Amersham Biosciences) and exposed to Biomax L film (Kodak). For quantification, ECL signals were digitized using Labworks software (UVP).

### 2.9. Immunohistochemical and Immunofluorescent Studies

The procedures and protocols for immunohistochemical (IHC) and immunofluorescent (IF) examinations were based on previous studies [31, 32]. Briefly, for IHC staining, rehydrated paraffin sections were first treated with 3% H_2_O_2_ for 30 minutes and incubated with Immuno-Block reagent (Bio SB) for 30 minutes at room temperature. Sections were then incubated with primary antibodies specifically against CD14 (1 : 300, Bio SS) at 4°C overnight. The irrelevant antibody [p53 (1 : 500, Abcam) and mouse control IgG (Abcam)] provided controls. IHC/IF stains were performed for Sca-1 (1 : 300, BioLegend), c-Kit (1 : 300, Santa Cruz), CD31 (1 : 100, Serotec), vWF (1 : 200, Millipore), SDF-1*α* (1 : 100, Santa Cruz), CXCR4 (1 : 200, Bioss), *γ*-H2AX (1 : 500, Abcam), CD68 (1 : 100, Abcam), TUNEL assay (In Situ Cell Death Detection Kit, Roche), XRCC1 (1 : 200, Abcam), cytochrome C (0.25 *μ*g/ml, Abcam), mitotracker (150 ng/ml, Molecular Probes®), and 53BP1 (0.25 *μ*g/ml, NOVUS) using respective primary antibodies and with irrelevant antibodies as controls. Three sections of heart specimens were analyzed per rat. For quantification, three randomly selected HPFs (×200 or 400x for IHC and IF studies) were analyzed for each section. The mean number per HPF for each animal was determined by summation of all numbers divided by 9.

### 2.10. Vessel Density in the LV Infarct Area

The procedure and protocol were as previously reported [29, 30]. IHC staining of small blood vessels was performed with *α*-SMA (1 : 400) as the primary antibody at room temperature for 1 hour, followed by washing with PBS thrice. Ten minutes after the addition of the anti-mouse-HRP conjugated secondary antibody, the tissue sections were washed with PBS thrice. Then 3,3′ diaminobenzidine (DAB) (0.7 g/tablet) (Sigma) was added, followed by washing with PBS thrice after one minute. Finally, hematoxylin was added as a counterstain for nuclei, followed by washing twice with PBS after one minute. Three heart sections were analyzed per rat. For quantification, three randomly selected HPFs (200x) were analyzed per section. The mean number per HPF for each animal was determined by summation of all numbers divided by 9.

### 2.11. Histological Studies of Fibrosis, Collagen Deposition, and Infarct Areas

The procedure and protocol for histological studies of fibrosis, collagen deposition, and infarct areas have been described previously [29, 30]. Masson's trichrome and Sirius red stainings were used to study fibrosis and collagen deposition in LV myocardium and H&E stain to identify the infarct area. Three 4 *μ*m thick serial sections of LV myocardium were prepared by Cryostat (Leica CM3050S). The integrated area (*μ*m^2^) of fibrosis, infarction, or collagen deposition in each section was calculated using Image J software (National Institutes of Health). Three sections were quantified for each animal, and three randomly selected HPFs (100x) were analyzed in each section. The number of pixels in each diseased area per HPF was determined and then summated from the three HPFs. The mean pixel number per HPF for each animal was then determined by summating all pixel numbers and dividing by 9. The mean integrated area (*μ*m^2^) of fibrosis, infarction, or collagen deposition in LV myocardium per HPF was finally obtained.

### 2.12. Statistical Analysis

Quantitative data are expressed as means ± SD. Statistical analysis was adequately performed by ANOVA followed by the Bonferroni multiple comparison post hoc test. SAS statistical software for Windows version 8.2 (SAS Institute, Cary, NC, USA) was utilized. A probability value < 0.05 was considered statistically significant.

## 3. Results

### 3.1. Time Course of Transthoracic Echocardiographic Findings prior to and after AMI in Four Groups of Animals (Table 1)

By day 0, left ventricular end-diastolic diameter (LVEDd), left ventricular end-systolic diameter (LVESd), and left ventricular ejection fraction (LVEF) did not differ among the five groups.

By day 14 after AMI, LVEDd was significantly higher in AMI than in other four groups, significantly higher in AMI-ECSW and AMI-SVF than in SC and AMI-ECSW-SVF, but it showed no difference between the latter two groups or among AMI-ECSW, AMI-SVF, and AMI-ECSW-SVF. Additionally, LVESd was highest in AMI and lowest in SC, significantly higher in AMI-ECSW and AMI-SVF than in AMI-ECSW-SVF, but similar between AMI-ECSW and AMI-SVF. Conversely, LVEF expressed an opposite pattern to LVESd among the five groups.

By day 42 after AMI, LVEDd and LVESd were highest in AMI, lowest in SC, significantly higher in AMI-ECSW and AMI-SVF than in AMI-ECSW-SVF, and showed no difference between AMI-ECSW and AMI-SVF. Additionally, LVEF showed an opposite pattern to LVEDd and LVESd among the five groups.

### 3.2. Flow Cytometric Analysis for Identification of SVF-Contained Cell Surface and Fluorescent Intensity of Mitotracker Staining in H9C2 Cells with and without Menadione and SVF Treatment (Figure 1)

Flow cytometry was used to analyze for adipose-derived SVF cellular components, including endothelial progenitor cell (EPC), cardiac stem cell, and mesenchymal stem cell (MSC) surface markers. The results showed that CD31 and CD29 were the most popular EPC and MSC surface makers of SVF, respectively.

To elucidate whether adipose-derived SVF treatment could prevent menadione-induced downregulated expression of mitochondria, the H9C2 cells (2 × 10^5^) were cocultured with freshly isolated adipose-derived SVF (1 × 10^5^ cells) for 24 h, followed by menadione (25 *μ*M) treatment for 30 minutes, then collected the cells for individual study. The results of IF microscopic finding showed that in a dose of 25 *μ*M menadione treatment for 30 minutes, the number of mitotracker emission positive cells (i.e., indicated mitochondrial mass) was notably reduced as compared with the control group. On the other hand, after 24 h pretreatment with SVF, the number of mitotracker emission positive cells was significantly increased regardless of with or without menadione treatment, suggesting mitochondrial numbers was enhanced in H9C2 cells after SVF treatment.

### 3.3. Coculture with SVF Reduced 53BP1 Nuclear Distribution in H9C2 Cells Undergoing Menadione Treatment (Figure 2)

The IF microscopic finding demonstrated that 53BP1 protein (i.e., an indicator of a double-stranded DNA damage marker) was enriched in the nuclei of H9C2 cells undergoing menadione treatment as compared with those of the control cells. However, this enhanced expression of 53BP1 protein in nuclei by menadione was remarkably reduced undergoing the SVF treatment (i.e., in situation of H9C2 cells cocultured with menadione-SVF). Additionally, fluorescent intensity of 53BP1+ nuclei was significantly higher in the menadione-treated group than in control and SVF-treated groups, suggesting that SVF prevented menadione-induced double-stranded DNA damage.

### 3.4. Coculture with SVF Decreased XRCC1 Nuclear Translocation in H9C2 Cells Undergoing Menadione Treatment and Identification of Dynamically Morphological Change of Fusion and Fission after Menadione Treatment (Figure 3)

The IF microscopic finding demonstrates that XRCC1 protein (i.e., another indicator of the double-stranded DNA damage marker) was enriched in the nuclei under menadione treatment as compared with the control cells. On one hand, in condition of H9C2 cells cocultured with AD-SVF, the distribution of XRCC1 in cytosol was substantially promoted. Additionally, the immunofluorescent intensity of XRCC1 in nuclei was significantly enhanced in the menadione-treated group than in the control that was significantly reversed in SVF treatment. On the other hand, as compared with the control group, the shrinkage of nuclear size was significantly enhanced in menadione-treated group that was significantly reversed by coculture with SVF.

The dynamically morphological change of mitochondrial punctate (i.e., indicated dynamics of mitochondria, including fusion and fission) was identified to be present in H9C2 cells after menadione treatment. As expected, the time courses (i.e., from 10 and 30 to 60 minutes) of mitochondrial punctate which was identified and found to be significantly and progressively increased after menadione treatment.

### 3.5. Condensed Collagen Deposition Area, Expression of Apoptotic Nuclei, and Histopathological Findings of Infarct Area and Fibrotic Area in LV Myocardium by Day 42 after AMI Induction (Figures 4 and 5)

**Figure 4:**
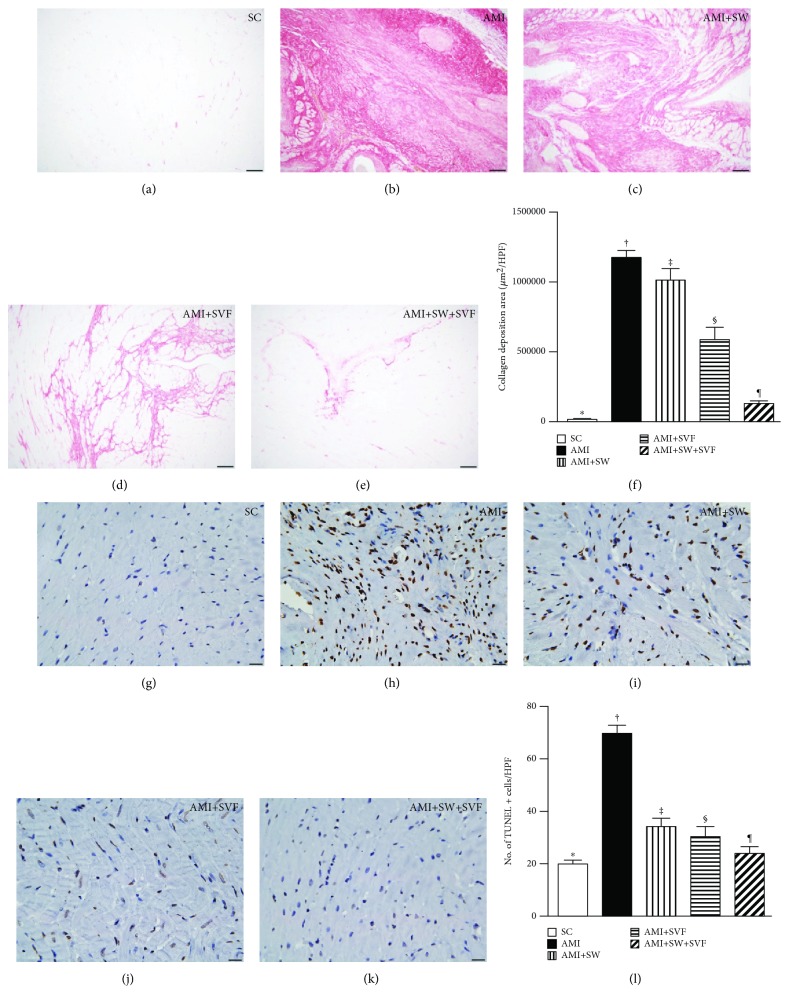
Condensed collagen deposition area and apoptotic nuclei in LV myocardium by day 42 after AMI induction. (a–e) Sirius stain microscopy (200x, red) to identify the condensed collagen deposition area. (f) Analysis of collagen deposition area, ^∗^ vs. other groups with different symbols (‡, ‡, §, ¶), *p* < 0.0001. (g–k) Immunohistochemistry (IHC, 100x, TUNEL assay) for apoptotic nuclei (gray). (l) Analysis of number of apoptotic nuclei, ^∗^ vs. other groups with different symbols (‡, ‡, §, ¶), *p* < 0.0001. All statistical analyses were performed by one-way ANOVA, followed by the Bonferroni multiple comparison post hoc test (*n* = 6 for each group). Symbols (^∗^, †, ‡, §) indicate significance (at 0.05 level). HPF = high-power field; SC = sham control; AMI = acute myocardial infarction; SW = shock wave; SVF = adipose-derived stromal vascular fraction.

IHC microscopy using Sirius stain demonstrated that the condensed collagen deposition area, an indicator of increased cardiomyocyte death and fibroblast activity, was largest in AMI, smallest in SC, significantly larger in AMI-ECSW and AMI-SVF than in AMI-ECSW-SVF and significantly larger in AMI-ECSW than in AMI-SVF (Figure 4). Additionally, the terminal deoxynucleotidyl transferase dUTP nick end labeling (TUNEL) assay showed that the number of apoptotic nuclei+ cells, indicators of cellular apoptosis, expressed an identical pattern to condensed collagen deposition area among the five groups (Figure 4).

**Figure 5:**
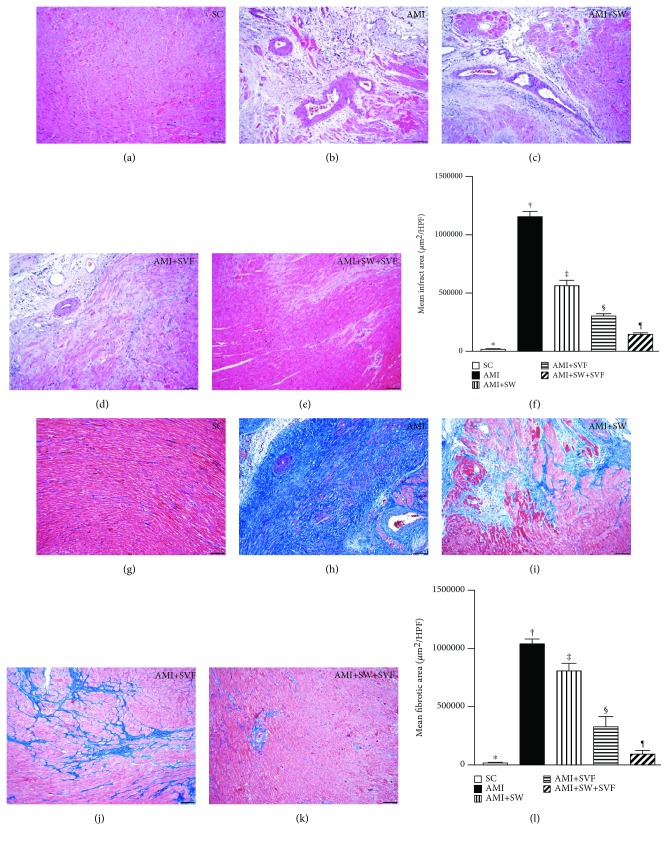
Histopathology for infarcted and fibrotic areas in LV myocardium by day 42 after AMI induction. (a–e) H&E staining (100x) to identify the infarct area. (f) Analysis of the infarct area, ^∗^ vs. other groups with different symbols (‡, ‡, §, ¶) , *p* < 0.0001. (g–k) Masson's trichrome stain (400x) to identify the fibrotic area in LV myocardium. (l) Analysis of the fibrotic area, ^∗^ vs. other groups with different symbols (‡, ‡, §, ¶), *p* < 0.0001. All statistical analyses were performed by one-way ANOVA, followed by the Bonferroni multiple comparison post hoc test (*n* = 6 for each group). Symbols (^∗^, †, ‡, §) indicate significance (at 0.05 level). HPF = high-power field; SC = sham control; AMI = acute myocardial infarction; SW = shock wave; SVF = adipose-derived stromal vascular fraction.

Furthermore, H&E staining and Masson's trichrome stain demonstrated that the infarct area and fibrotic area coincided with the collagen deposition area among the five groups, respectively (Figure 5).

### 3.6. Identifications of Cardiac Stem Cells, Endothelial Cells, and Endothelial Progenitor Cells in LV Myocardium by Day 42 after AMI Induction (Figures 6 [Fig fig7]–8)

**Figure 6:**
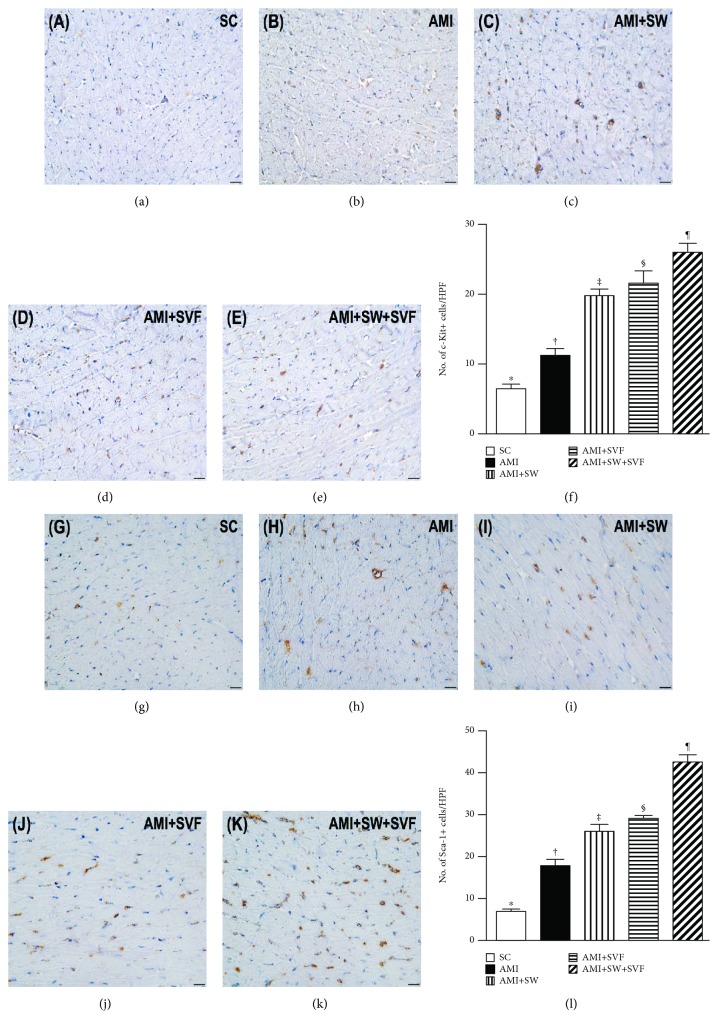
Identification of cardiac stem cells in LV myocardium by day 42 after AMI induction. (a–e) IHC (400x) to identify c-Kit+ cells (gray). (f) Analysis of number of c-Kit+ cells, ^∗^ vs. other groups with different symbols (‡, ‡, §, ¶), *p* < 0.0001. (g–k) IHC microscopy (400x) for Sca-1+ cells (gray). (l) Analysis of number of Sca-1+ cells, ^∗^ vs. other groups with different symbols (‡, ‡, §, ¶), *p* < 0.0001. All statistical analyses were performed by one-way ANOVA, followed by the Bonferroni multiple comparison post hoc test (*n* = 6 for each group). Symbols (^∗^, †, ‡, §) indicate significance (at 0.05 level). HPF = high-power field; SC = sham control; AMI = acute myocardial infarction; SW = shock wave; SVF = adipose-derived stromal vascular fraction.

IHC microscopy established that the number of positively stained c-Kit and positively stained Sca-1 cells, two indicators of cardiac stem cells, progressively increased from SC to AMI-ECSW-SVF (Figure 6), suggesting that increased c-Kit+ cells in LV myocardium were an intrinsic response to ischemic stimulation that was further increased by ECSW-SVF treatment.

**Figure 7 fig7:**
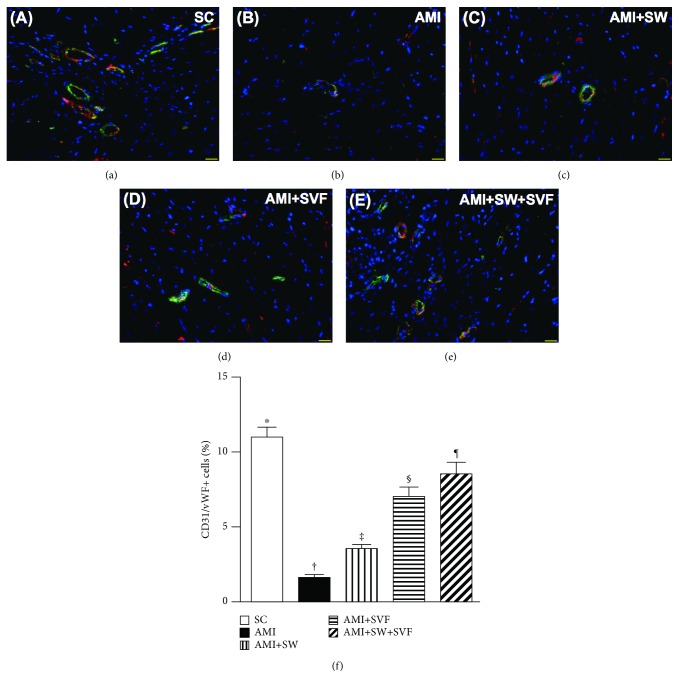
Expression of endothelial cells in LV myocardium by day 42 after AMI induction. (a–e) Immunofluorescence (IF; 400x) to identify CD31+ cells (green). (f) Analysis of numbers of CD31+ cells, ^∗^ vs. other groups with different symbols (‡, ‡, §, ¶), *p* < 0.0001. (g–k) IF microscopy (400x) for the von Willebrand factor+ (vWF+) cells (green). (l) Analysis for the number of vWF+ cells, ^∗^ vs. other groups with different symbols (‡, ‡, §, ¶), *p* < 0.0001. Red color in (d, e, j, and k) indicates the implanted MSCs in LV myocardium. All statistical analyses were performed by one-way ANOVA, followed by the Bonferroni multiple comparison post hoc test (*n* = 6 for each group). Symbols (^∗^, †, ‡, §) indicate significance (at 0.05 level). HPF = high-power field; SC = sham control; AMI = acute myocardial infarction; SW = shock wave; SVF = adipose-derived stromal vascular fraction.

**Figure 8:**
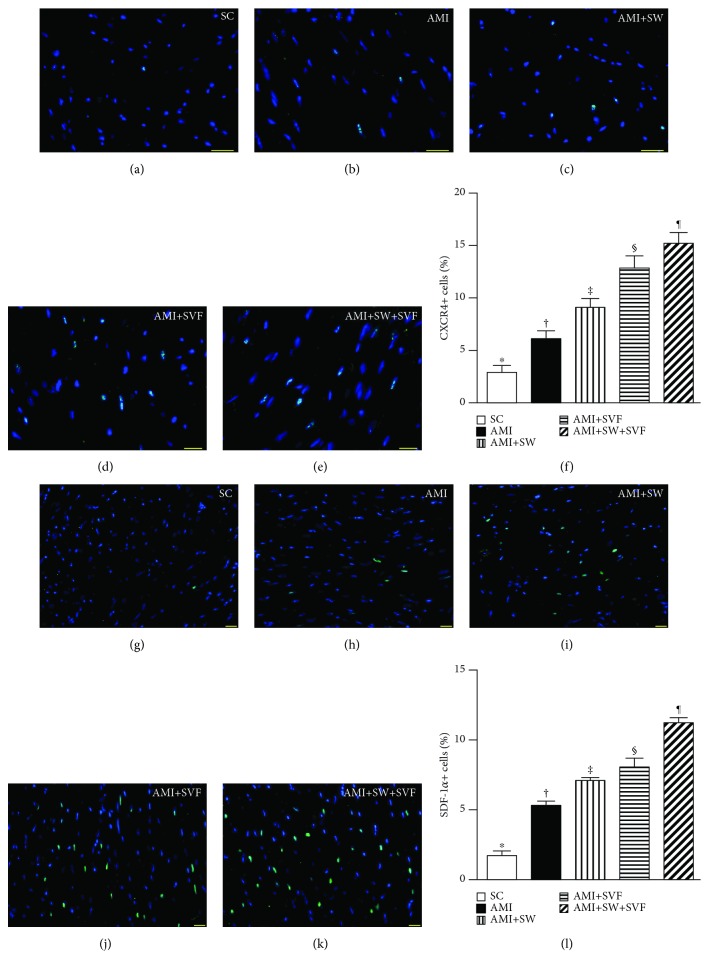
Expression of endothelial progenitor cells in LV myocardium by day 42 after AMI induction. (a–e) IF microscopy (400x) to identify CXCR4+ cells (green). (f) Analysis for the number of CXCR4+ cells, ^∗^ vs. other groups with different symbols (‡, ‡, §, ¶), *p* < 0.0001. (g–k) IF microscopy (400x) to identify stromal cell-derived factor- (SDF-) 1*α*+ cells (green color). (l) Analysis of the number of SDF-1*α*+ cells, ^∗^ vs. other groups with different symbols (‡, ‡, §, ¶), *p* < 0.0001. Red color in (d, e, j, and k) indicates the implanted MSCs in LV myocardium. All statistical analyses were performed by one-way ANOVA, followed by the Bonferroni multiple comparison post hoc test (*n* = 6 for each group). Symbols (^∗^, †, ‡, §) indicate significance (at 0.05 level). HPF = high-power field; SC = sham control; AMI = acute myocardial infarction; SW = shock wave; SVF = adipose-derived stromal vascular fraction.

Additionally, IF microscopy showed that the numbers of CD31+ and von Willebrand factor+ (vWF+) cells, two indicators of endothelial cells, were highest in SC and lowest in AMI, significantly lower in AMI-ECSW and AMI-SVF than in AMI-ECSW-SVF, and significantly lower in AMI-ECSW than in AMI-SVF (Figure 7). On the other hand, IF microscopy also revealed that the numbers of C-X-C motif chemokine receptor 4 (CXCR4)+ and stromal cell-derived factor (SDF)-1*α*+ cells progressively increased from SC to AMI-ECSW-SVF (Figure 8), also suggesting an intrinsic response to ischemic stimulation and enhanced expression by ECSW-SVF treatment.

### 3.7. Immunofluorescent Staining for DNA Damage and Inflammatory Biomarkers in LV Myocardium by Day 42 after AMI Induction (Figure 9)

IF microscopy displayed that the number of *γ*-H2AX+ cells, indicators of DNA damage, was highest in AMI, lowest in SC, significantly higher in AMI-ECSW and AMI-SVF than in AMI-ECSW-SVF, and significantly higher in AMI-ECSW than in AMI-SVF. Additionally, IF microscopy exhibited that the number of CD68+ cells, indices of inflammation, expressed an identical pattern to *γ*-H2AX among the five groups.

### 3.8. Protein Expression of Angiogenesis Biomarkers in LV Myocardium by Day 42 after AMI Induction (Figure 10)

The protein expressions of endothelial nitric oxide synthase (eNOS) and CD31, two biomarkers indicating integrity of endothelial cell/angiogenesis, were highest in SC, lowest in AMI, significantly higher in AMI-ECSW-SVF than in AMI-ECSW and AMI-SVF, and significantly higher in AMI-SVF than in AMI-ECSW. Protein expression of vascular endothelial growth factor (VEGF), another indicator of integrity of endothelial cell/angiogenesis biomarker, progressively increased from SC to AMI-ECSW-SVF. The protein expressions of CXCR4 and SDF-1*α*, two indicators of endothelial progenitor cell/angiogenesis biomarkers, showed an identical pattern to VEGF among the five groups.

### 3.9. Protein Expressions of Inflammatory, Oxidative Stress, Energy Indicators, Apoptotic, and Fibrosis Biomarkers in LV Myocardium by Day 42 after AMI Induction (Figures 11 and 12)

**Figure 11:**
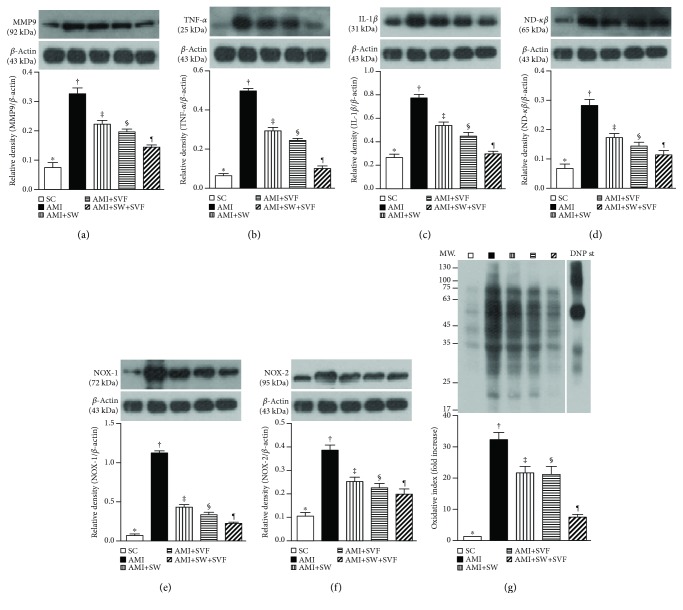
Protein expression of inflammation and oxidative stress biomarkers in LV myocardium by day 42 after AMI induction. Protein expressions of (a) matrix metalloproteinase- (MMP-) 9, (b) tumor necrotic factor- (TNF-) *α*, (c) interleukin- (IL-) 1*β*, (d) nuclear factor- (NF-) *κ*B, (e) NOX-1, (f) NOX-2, ^∗^ vs. other groups with different symbols (‡, ‡, §, ¶), *p* < 0.001, and (g) oxidized protein expression, ^∗^ vs. other groups with different symbols (†, ‡, §, ¶), *p* < 0.0001. (Note: left and right lanes shown on the upper panel represent protein molecular weight marker and control oxidized molecular protein standard, respectively). M.W. = molecular weight; DNP = 1-3 dinitrophenylhydrazone. All statistical analyses were performed by one-way ANOVA, followed by Bonferroni multiple comparison post hoc test (*n* = 6 for each group). Symbols (^∗^, †, ‡, §) indicate significance (at 0.05 level). SC = sham control; AMI = acute myocardial infarction; SW = shock wave; SVF = adipose-derived stromal vascular fraction.

The protein expressions of matrix metalloproteinase (MMP)-9, tumor necrosis factor- (TNF-) *α*, interleukin- (IL-) 1*β*, and nuclear factor- (NF-) *κ*B, four indices of inflammation, were highest in AMI, lowest in SC, significantly higher in AMI-ECSW and AMI-SVF than in AMI-ECSW-SVF, and significantly higher in AMI-ECSW than in AMI-SVF (Figure 11). Additionally, protein expressions of NADPH oxidase- (NOX-) 1, NOX-2, and oxidized protein, three indices of oxidative stress, showed an identical pattern to inflammatory biomarkers among the five groups (Figure 11).

**Figure 12:**
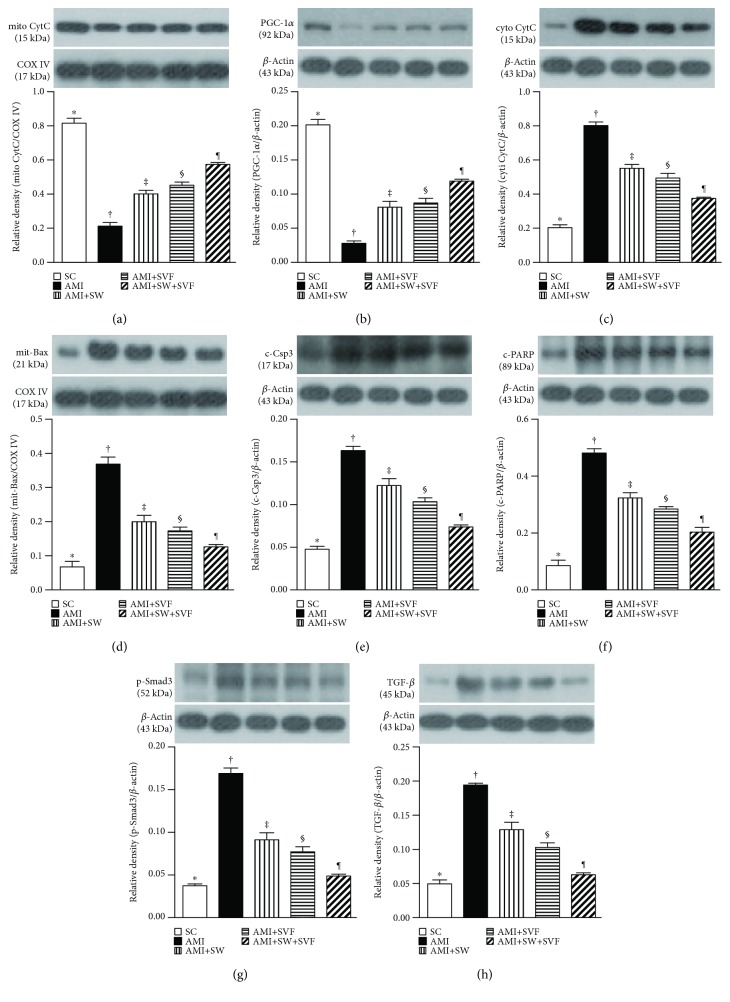
Protein expressions of energy reservoirs, apoptotic, and fibrosis biomarkers in LV myocardium by day 42 after AMI induction. Protein expression of (a) mitochondrial cytochrome C (mit-Cyt C), (b) peroxisome proliferator activated receptor-*γ* coactivator 1 *α* (PGC-1*α*), (c) cytosolic cytochrome C (cyt-Cyt C), (d) mitochondrial Bax (mit-Bax), (e) cleaved caspase 3 (c-Casp 3), (f) cleaved poly(ADP-ribose) polymerase (c-PARP), (g) Smad3, and (h) transforming growth factor- (TGF-) *β*, ^∗^ vs. other groups with different symbols (†, ‡, §, ¶), *p* < 0.0001. All statistical analyses were performed by one-way ANOVA, followed by the Bonferroni multiple comparison post hoc test (*n* = 6 for each group). Symbols (^∗^, †, ‡, §) indicate significance (at 0.05 level). SC = sham control; AMI = acute myocardial infarction; ECSW = shock wave; SVF = adipose-derived stromal vascular fraction.

On the other hand, the protein expressions of mitochondrial cytochrome C, an indicator of mitochondrial integrity and PCG1-*α*, an indicator of energy biogenesis promotor, exhibited an opposite pattern to inflammation among the five groups (Figure 12). However, the protein expression of cytosolic cytochrome C, an indicator of mitochondrial damage, exhibited an opposite pattern to PCG1-*α* among the five groups (Figure 12).

The protein expressions of mitochondrial Bax, cleaved caspase 3, and cleaved poly(ADP-ribose) polymerase (PARP), three apoptotic biomarkers, showed an identical pattern to cytosolic cytochrome C among the five groups. Additionally, the protein expressions of Smad3 and transforming growth factor- (TGF-) *β*, two indicators of fibrosis, displayed an identical pattern to apoptosis among the five groups.

### 3.10. Protein Expressions of DNA Damage, Pressure Overload/Heart Failure Biomarkers, and Small Vessel Density and Muscularization in the LV Infarct Area by Day 42 after AMI Induction (Figure 13)

The protein expression of *γ*-H2AX, an indicator of DNA damage, was highest in AMI, lowest in SC, significantly higher in AMI-ECSW and AMI-SVF than in AMI-ECSW-SVF, and significantly higher in AMI-ECSW than in AMI-SVF. Protein expression of brain natriuretic peptide (BNP), a biomarker for heart failure, showed an identical pattern to *γ*-H2AX among the five groups. Protein expression of *β*-myosin heavy chain (MHC), an indicator of myocardial hypertrophy, displayed an identical pattern to BNP among the five groups. However, protein expression of *α*-MHC, a reverse myocardial hypertrophy biomarker, exhibited an opposite pattern to *β*-MHC among the five groups.

The number of small vessels (<25 *μ*M) in the infarct area was highest in SC, lowest in AMI, and significantly progress from AMI-ECSW to AMI-ECSW-SVF.

### 3.11. 2,3,5-Triphenyltetrazolium Chloride (TTC) Staining for Identification of LV Myocardial Infarction Area by Day 14 after AMI Induction (Supplemental Figure 1)

The TTC stain identified that the LV myocardial infarction area was highest in group 2, smallest in group 1, significantly larger in groups 3 and 4 than in group 5, and significantly larger in group 3 than in group 4.

## 4. Discussion

An essential finding in the present study was that the infarction area, fibrotic area, and condensed collagen deposition area were significantly smaller in AMI-ECSW and AMI-SVF groups than in AMI groups. These findings could explain the most important finding of the present study that the LVEF was significantly higher in AMI-ECSW and AMI-SVF animals than in AMI animals. Additionally, the LVEDd and LVESd parameters were remarkably higher in the AMI group than in the control, suggesting that LV remodeling inevitably develops after AMI. However, these two parameters were reduced in AMI animals treated by ECSW and SVF. Intriguingly, our previous studies have shown that ECSW [24] and MSC [9, 10, 29, 30] therapy significantly improved LV function and markedly attenuated LV remodeling in rodent and swine AMI models. Accordingly, the results of the present study were consistent with those of previous studies [9, 10, 24, 29, 30]. Of particular importance was that combined ECSW-SVF therapy was superior to either one for reducing LV infarct size and preserving heart function after AMI. Our findings, therefore, extended the findings of previous work [9, 10, 24, 29, 30].

An important finding in the in vitro study was that the fluorescent intensity of 53BP1-positively stained protein in nuclei of H9C2 cells was significantly enhanced/condensed in the menadione-treated group as compared with the control group. However, this phenomenon was reversed in menadione-treated H9C2 cells after SVF treatment. Additionally, the cytoplasmic distribution of XRCC1-positively stained H9C2 cells was markedly reduced whereas the fluorescent intensity of XRCC1 in nuclei was notably increased in the menadione-treated group compared with the control group. However, this phenomenon was attenuated in menadione-treated H9C2 cells followed by SVF treatment. Furthermore, the results of in vitro study demonstrated that the fluorescent intensity of mitotracker-positively stained nuclei (i.e., an index of mitochondria) in menadione-treated H9C2 cells was significantly reduced, compared with the control group, and this was reversed after SVF treatment. In vivo study showed that DNA damage (i.e., *γ*-H2AX) and mitochondrial damage (cytosolic cytochrome C) cells were significantly increased in AMI animals than in SC animals, were substantially reduced after receiving SVF or ECSW treatment, and were further substantially reduced after receiving combined ECSW-SVF treatment. This is consistent with previous studies which established that ECSW-MSC therapy inhibited the expressions of DNA damage and mitochondrial damage biomarkers in rodent and swine AMI models [6, 9, 24, 30]. Accordingly, the results of our in vitro and in vivo studies, in addition to strengthening the findings of previous studies [6, 9, 24, 30], could, at least in part, explain why ECSW-supported SVF significantly preserved LV function and ameliorated LV remodeling.

Another important finding of the present study was that ECSW and SVF were comparable for significant inhibitions of inflammatory reaction, fibrosis, apoptosis, and generation of oxidative stress. Interestingly, experimental studies have previously shown that ECSW and MSC therapy significantly improved ischemia-related organ dysfunction mainly through suppressing the inflammatory reaction, cellular apoptosis, and the generation of oxidative stress [6, 11, 12, 16, 30, 33]. In this way, our present findings corroborate those of previous studies [6, 11, 12, 16, 30, 33]. Of importance was that combined ECSW-SVF therapy was superior to either ECSW or SVF alone for suppressing these parameters, again partially explaining why ECSW-SVF combination therapy was superior to either alone for improving outcomes in rats after AMI. Accordingly, our preclinical findings suggest that combined ECSW-SVF could potentially be a therapeutic option for AMI patients who are refractory to conventional therapy.

LV remodeling-associated LV hypertrophy and upregulation of pressure overload/heart failure biomarkers are essential findings in ischemic cardiovascular syndrome [32–34]. Additionally, PGC-1*α*, which is involved in the regulation of oxidative metabolism and mitochondrial biogenesis, was downregulated in congestive heart failure and AMI [35, 36]. A principal finding in the present study was that the protein expressions of BNP and *β*-MHC were significantly higher, whereas the protein expression of PGC-1*α* was notably lower, in AMI animals than in SC animals. Thus, these findings could, at least in part, explain why LVEF was remarkably reduced and LVEDs/LVESd were substantially increased in AMI animals compared to SC animals. Of particular importance was that these parameters were reversed in AMI animals after receiving ECSW or SVF therapy and were further altered in AMI animals after receiving combined ECSW-SVF therapy.

Interestingly, when we looked at the flow cytometric result of SCF components, we found that the percentage of cardiac progenitor cells was very low in the SVF. However, IHC microscopic findings of LV myocardium showed that the number of cardiac progenitor cells (i.e., Sca-1+, c-Kit+ cells) was notably much higher in SVF-ECSW therapy animals than in SC animals. In general, it seems too difficult for the implanted cardiac progenitor cells to retain in the infarct area for a long time. Accordingly, we propose that the majority of these cardiac progenitor cells might be a result of homing from circulation and bone marrow to the infarct area of LV myocardium.

### 4.1. Study Limitations

This study has some limitations. First, stepwise increases in the dosages of ECSW and SVF were not performed in the present study. Thus, optimal dosing for ECSW and SVF was not verified. Second, although extensive work was done and the results of the present study were promising, the exact mechanisms underlying preservation of heart function after ECSW-SVF treatment of AMI are still largely unclear.

## 5. Conclusions

In conclusion, the present study demonstrated that ECSW-SVF therapy effectively preserved LV function and inhibited LV remodeling mainly via the attenuation of inflammation, apoptosis, oxidative stress, and DNA damage and the augmentation of angiogenesis.

## Figures and Tables

**Figure 1:**
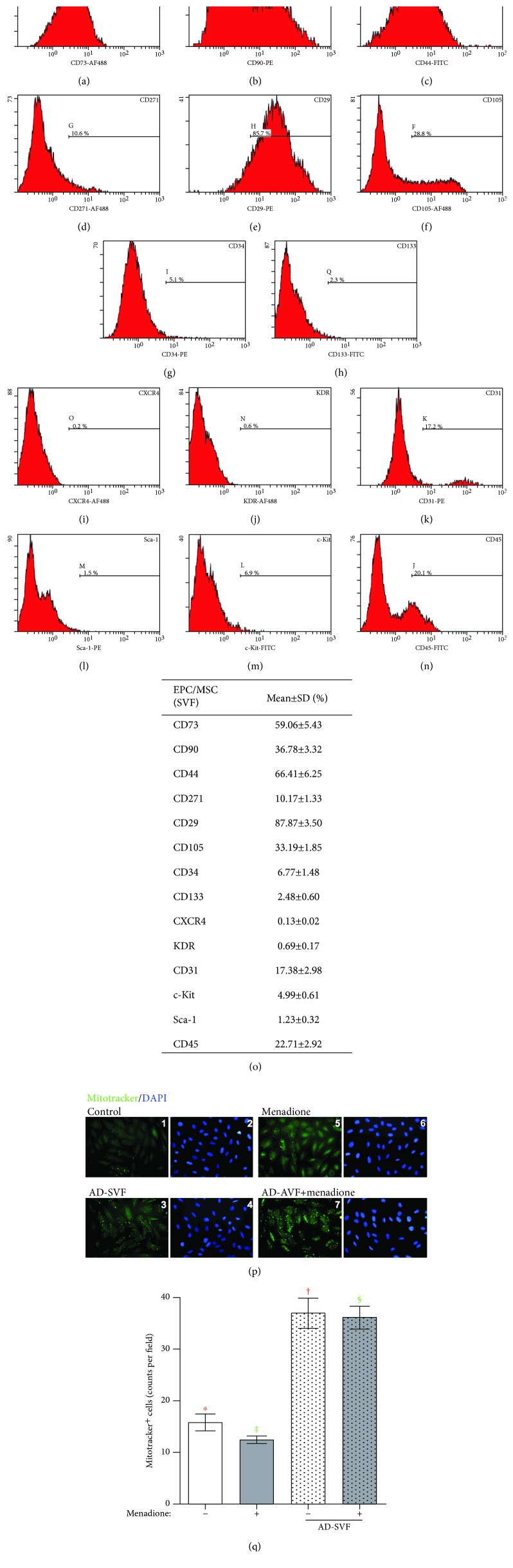
Surface markers of EPC and MSC and fluorescent intensity of mitotracker staining in H9C2 cells undergoing menadione and SVF treatments. (a–n) Flow cytometry for endothelial progenitor cell (EPC), cardiac stem cell, and mesenchymal stem cell (MSC) surface markers. (o) Flow cytometric analysis (*n* = 6) showed that CD34 and CD271 were the clearest EPC and MSC surface markers for SVF, respectively. (p, 1–8) Illustrating the immunofluorescent (IF) microscopic finding of the mitotracker stain for identification of mitochondria in H9C2 cells in the normal control group (p, 1 and 2) and AD-SVF treated group (p, 3 and 4). The number of mitotracker emission positive cells (i.e., counted in five randomly chosen fields) was significantly increased after receiving SVF treatment, ^∗^ vs. †, *p* < 0.0001 (red symbols). (p, 5–8) Illustrating the IF microscopic finding of the mitotracker stain for identification of mitochondria in H9C2 cells in the menadione-treated group (p, 5 and 6) and menadione + AD-SVF treated group (p, 7 and 8). The number of mitotracker emission positive cells was significantly increased in menadione + SVF treatment than in menadione only, ‡ vs. §, *p* < 0.001 (green symbols). Blue color indicated nuclei stained by DAPI. *n* = 4 in each group. AD-SVF = adipose-derived stromal vascular fraction.

**Figure 2:**
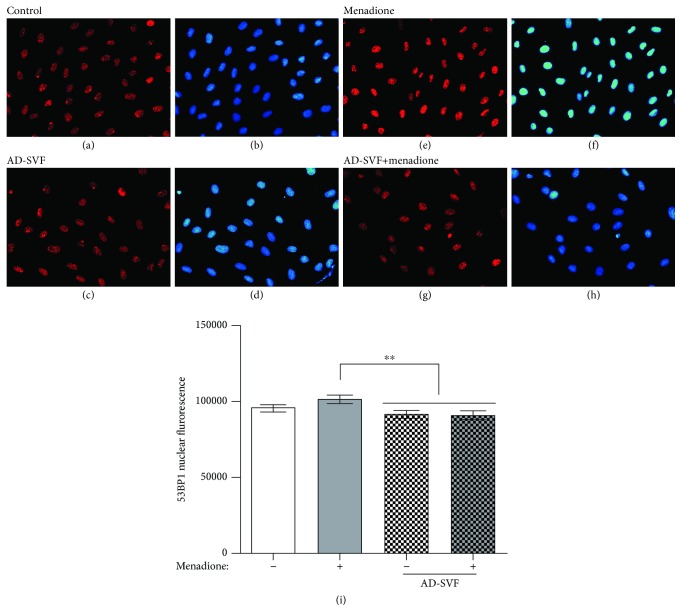
Coculture with SVF reduced 53BP1 nuclear distribution in H9C2 cells undergoing menadione treatment. (a–h) Illustrating the immunofluorescent (IF) microscopic finding for identification of positively stained 53BP1 in nuclei of the H9C2 cell with and without menadione and SVF treatment. The results showed that the 53BP1 protein was enriched in the nuclei of H9C2 cells undergoing menadione treatment as compared with those of the control cells (a vs. e). However, this enhanced expression of 53BP1 protein in nuclei by menadione was remarkably reduced undergoing the SVF treatment (e vs. g). (i) Nuclear fluorescent intensity of 53BP1 was calculated by Image J software from 5 random fields (*n* = 4/each group). Data were expressed as means ± SEM. ^∗∗^ indicated *p* < 0.01. AD-SVF = adipose-derived stromal vascular fraction.

**Figure 3:**
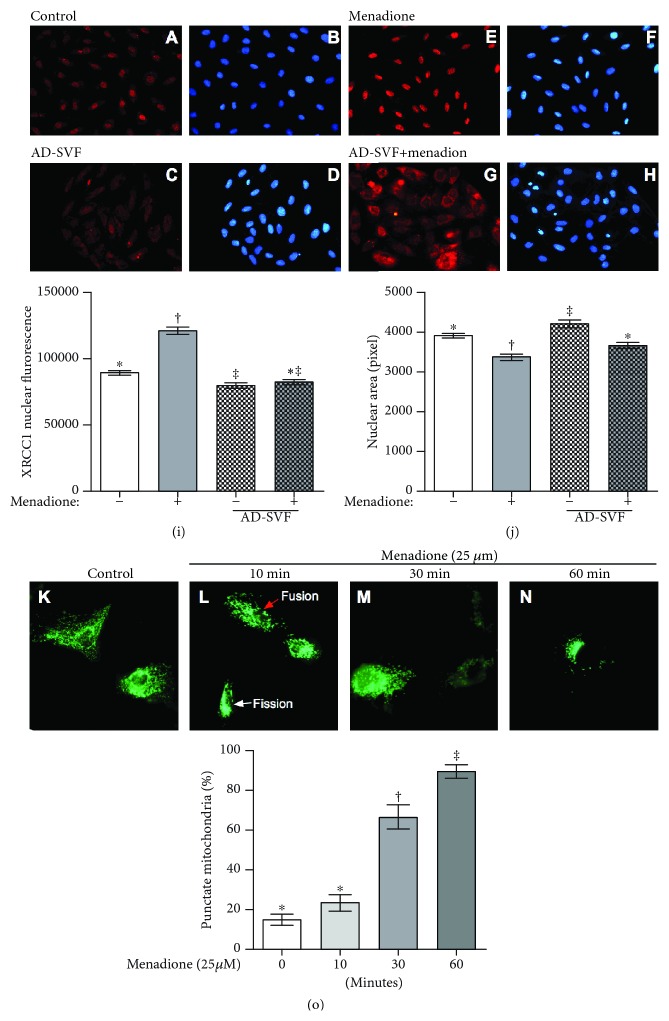
Coculture with SVF decreased XRCC1 nuclear translocation in H9C2 cells undergoing menadione treatment and identification of fusion and fission after menadione treatment. (a–h) Illustrating the immunofluorescent (IF) microscopic finding for identification of positively stained XRCC1 protein in nuclei of the H9C2 cell with and without menadione and SVF treatment. The results showed that XRCC1 protein was notably enriched in the nuclei under menadione treatment (e) as compared with the control cells (a). On the other hand, however, in condition of H9C2 cells cocultured with AD-SVF, the distribution of XRCC1 in cytosol was notably promoted (g). (i) Fluorescent intensity of 53BP1 in nuclei was calculated by Image J software from 5 random fields (*n* = 4/each group). Analytical result: ^∗^ vs. other groups with different symbols (†, ‡), *p* < 0.001. (j) Nuclear area was calculated by Image J software from 5 random fields (*n* = 4/each group). Analytical result: ^∗^ vs. other groups with different symbols (†, ‡), *p* < 0.001. AD-SVF = adipose-derived stromal vascular fraction. (k–n) Illustrating the time courses of menadione induced the process of mitochondrial fusion/fission (from 0, 10, and 30 to 60 minutes), i.e., monitored by mitochondrial-targeted EGFP fluorescence protein. The cellular apoptosis of MT-EGFP transfected into H9C2 cells was observed after menadione (25 *μ*M) administration. The morphological change of mitochondria (i.e., punctate appearance) was identified by fluorescence microscope Olympus BX51 (Olympus America Inc., Melville, NY, USA). Red arrow indicated mitochondrial morphology of fusion, and the white arrow indicated mitochondrial morphology of fission. (o) Analytic results of proportion of punctate mitochondria were counted over 100 cells, ^∗^ vs. other groups with different symbols (‡, ‡), *p* < 0.0001. Statistical analysis was performed by one-way ANOVA, followed by the Bonferroni multiple comparison post hoc test (*n* = 6 for each group). Symbols (^∗^, †, ‡) indicate significance (at 0.05 level).

**Figure 9:**
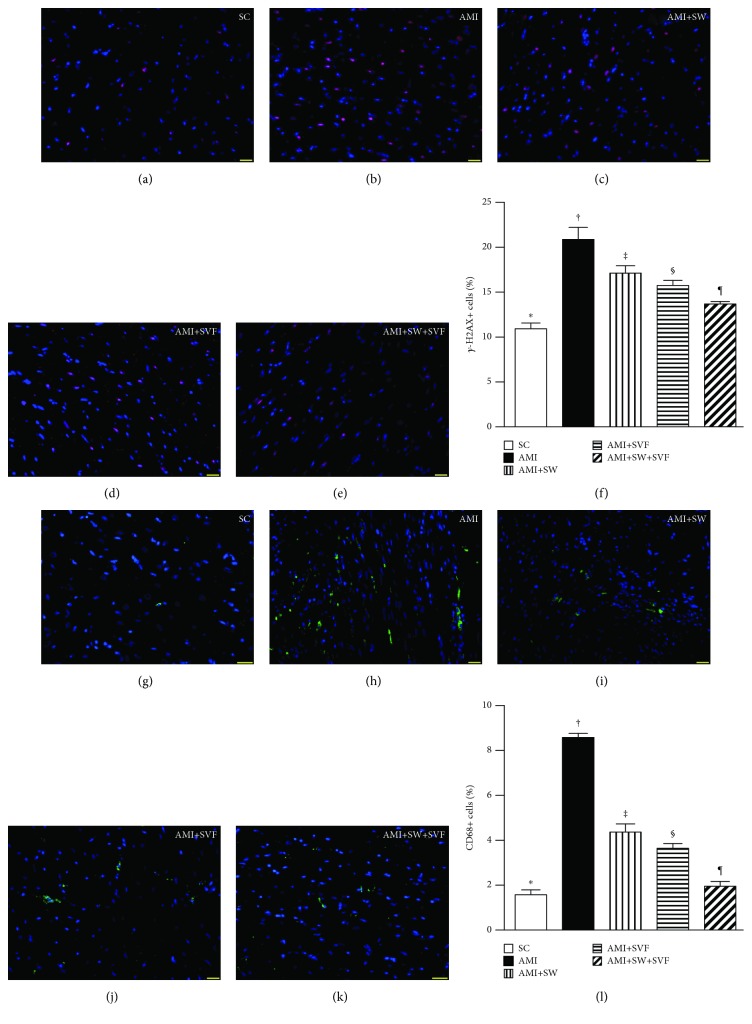
Identification of DNA damage and inflammatory biomarkers in LV myocardium by day 42 after AMI induction. (a–e) IF microscopy (400x) to identify *γ*-H2AX+ cells (green color). (f) Analysis of the number of *γ*-H2AX+ cells, ^∗^ vs. other groups with different symbols (‡, ‡, §, ¶), *p* < 0.0001. (g–k) IF microscopy for CD68+ cells (green color). Analysis of number of CD68+ cells, ^∗^ vs. other groups with different symbols (‡, ‡, §, ¶), *p* < 0.0001. Red color in (d, e, j, and k) indicates the implanted MSCs in LV myocardium. All statistical analyses were performed by one-way ANOVA, followed by the Bonferroni multiple comparison post hoc test (*n* = 6 for each group). HPF = high-power field; SC = sham control; AMI = acute myocardial infarction; SW = shock wave; SVF = adipose-derived stromal vascular fraction.

**Figure 10:**
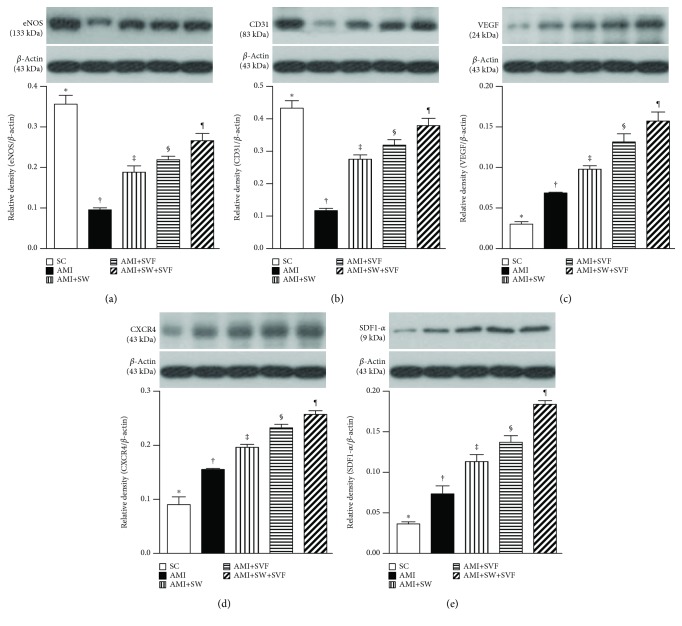
Protein expression of angiogenesis biomarkers in LV myocardium by day 42 after AMI induction. Protein expression of (a) endothelial nitric oxide synthase (eNOS), (b) CD31, (c) vascular endothelial growth factor (VEGF), (d) CXCR4, and (e) stromal cell-derived factor- (SDF-) 1*α*, ^∗^ vs. other groups with different symbols (‡, ‡, §, ¶), *p* < 0.001. All statistical analyses were performed by one-way ANOVA, followed by the Bonferroni multiple comparison post hoc test (*n* = 6 for each group). Symbols (^∗^, †, ‡, §) indicate significance (at 0.05 level). SC = sham control; AMI = acute myocardial infarction; SW = shock wave; SVF = adipose-derived stromal vascular fraction.

**Figure 13:**
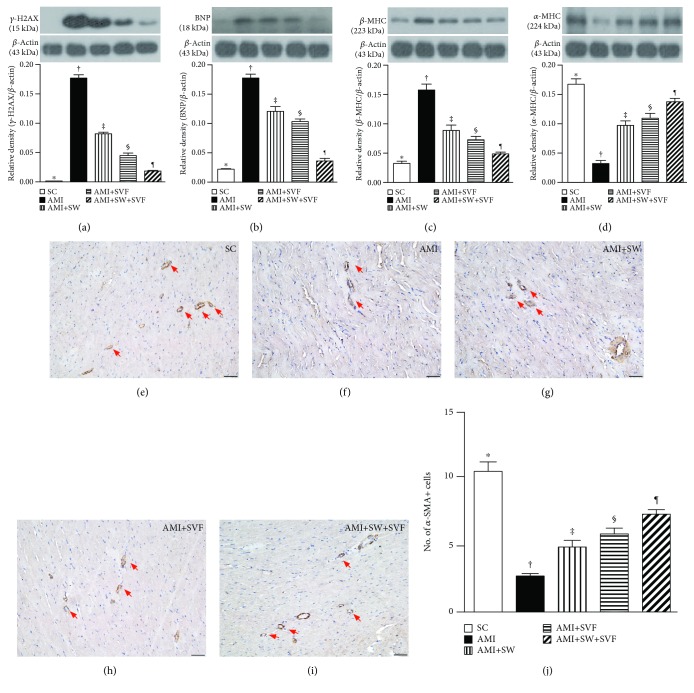
Protein expressions of DNA damage, pressure overload/heart failure biomarkers, and small vessel density in the LV infarct area by day 42 after AMI induction. Protein expression of (a) *γ*-H2AX, (b) brain natriuretic peptide (BNP), (c) *β*-myosin heavy chain (MHC), and (d) *α*-MHC, ^∗^ vs. other groups with different symbols (†, ‡, §, ¶), *p* < 0.0001. (e–i) Illustrating *α*-smooth muscle actin staining to identify the number of small vessels (<25 *μ*M, 100x, red arrows). (j) Analysis of number of small vessels (yellow arrows), ^∗^ vs. other groups with different symbols (†, ‡, §, ¶), *p* < 0.0001. All statistical analyses were performed by one-way ANOVA, followed by the Bonferroni multiple comparison post hoc test (*n* = 6 for each group). Symbols (^∗^, †, ‡, §) indicate significance (at 0.05 level). HPF = high-power field; SC = sham control; AMI = acute myocardial infarction; SW = shock wave; SVF = adipose-derived stromal vascular fraction.

**Table 1:** Time courses of echocardiographic findings among five groups.

Variables	Group 1 (*n* = 6)	Group 2 (*n* = 6)	Group 3 (*n* = 6)	Group 4 (*n* = 6)	Group 5 (*n* = 6)	*p* value^∗^
Day 0						
LVEDd (mm)	7.91 ± 0.33	7.86 ± 0.29	7.87 ± 0.27	7.88 ± 0.32	7.90 ± 0.39	0.882
LVESd (mm)	4.32 ± 0.32	4.36 ± 0.30	4.41 ± 0.21	4.29 ± 0.38	4.33 ± 0.30	0.741
LVEF (%)	72.98 ± 0.92	72.94 ± 0.85	73.3 ± 0.44	73.46 ± 1.03	73.22 ± 0.68	0.633
Day 14						
LVEDd (mm)	8.01 ± 0.31^a^	8.94 ± 0.36^b^	8.21 ± 0.22^c^	8.22 ± 0.23^c^	8.11 ± 0.26^a,c^	<0.001
LVESd (mm)	4.45 ± 0.30^a^	5.74 ± 0.24^b^	5.28 ± 0.19^c^	5.29 ± 0.21^c^	4.89 ± 0.23^d^	<0.0001
LVEF (%)	73.9 ± 0.88^a^	59.1 ± 1.02^b^	63.16 ± 0.99^c^	63.22 ± 1.01^c^	68.2 ± 1.03^d^	<0.0001
Day 42						
LVEDd (mm)	8.17 ± 0.28^a^	9.51 ± 0.46^b^	8.96 ± 0.32^c^	9.01 ± 0.29^c^	8.46 ± 0.34^d^	<0.0001
LVESd (mm)	4.44 ± 0.36^a^	6.41 ± 0.39^b^	5.52 ± 0.28^c^	5.49 ± 0.32^c^	4.83 ± 0.29^d^	<0.0001
LVEF (%)	74.2 ± 0.79^a^	57.6 ± 1.29^b^	66.43 ± 1.39^c^	66.21 ± 0.86^c^	70.2 ± 0.99^d^	<0.0001

Data are expressed as mean ± SD. Group 1 = sham control; group 2 = AMI; group 3 = AMI + ECSW; group 4 = AMI + SVF; group 5 = AMI + ECSW + SVF. AMI = acute myocardial infarction; ECSW = extracorporeal shock wave; SVF = adipose-derived stromal vascular fraction; LVEDd = left ventricular end-diastolic dimension; LVESd = left ventricular end-systolic dimension; LVEF = left ventricular ejection fraction. ^∗^ Letters (^a,b,c,d^) indicate significant difference (at 0.05 level) by Tukey multiple comparison procedure.

## Data Availability

The data used to support the findings of this study are available from the corresponding author upon request (han.gung@msa.hinet.net).

## Supplementary Materials

Supplementary Materials Supplemental Figure 1: 2,3,5-triphenyltetrazolium chloride (TTC) staining for identification of the LV myocardial infarction area by day 14 after AMI induction.
